# The interrelationship between periodontal disease and systemic health

**DOI:** 10.1038/s41415-025-8642-2

**Published:** 2025-07-25

**Authors:** Rajpal Tattar, Bruna Dias Cazvalho da Costa, Vitor C. M. Neves

**Affiliations:** 119776395004477103976 PhD Candidate, Restorative Dentistry Unit, The School of Clinical Dentistry, University of Sheffield, Sheffield, United Kingdom; 295282975226183045557 Senior Clinical Lecturer and Honorary Consultant in Periodontology, Restorative Dentistry Unit, The School of Clinical Dentistry, University of Sheffield, Sheffield, United Kingdom

## Abstract

**Background** Periodontal disease is a chronic inflammatory disease and has been linked to various systemic conditions. The chronic inflammation associated with periodontal disease could affect systemic health, while systemic conditions may also impact periodontal disease progression and severity.

**Aims** This narrative review aims to examine the associations between periodontal disease and systemic diseases, with a focus on understanding the underlying mechanisms for each bodily system.

**Methods** A comprehensive literature review was conducted to evaluate studies investigating periodontal disease as a potential factor influencing systemic health. Key topics included mechanisms of microbial translocation, inflammation and immune dysregulation across various systems.

**Results** Periodontal disease has been associated with systemic inflammation and immune responses that may contribute to the development or exacerbation of systemic conditions. Specific pathways may involve bacterial translocation, cytokine production and immune hyperresponsiveness, all of which may affect systemic health.

**Discussion** Managing periodontal health may reduce inflammatory markers associated with systemic diseases; although, evidence remains inconclusive for most conditions due to study heterogeneity.

**Conclusion** Understanding the interactions between periodontal disease and systemic conditions could guide integrated healthcare strategies, emphasising periodontal care to improve systemic health outcomes. Further research is needed to clarify causal relationships and therapeutic benefits.

**Supplementary Information:**

Zusatzmaterial online: Zu diesem Beitrag sind unter 10.1038/s41415-025-8642-2 für autorisierte Leser zusätzliche Dateien abrufbar.

## Key points

- Periodontal disease, a common inflammatory condition affecting tooth-supporting structures, is associated with numerous systemic conditions.
- Research highlights how periodontal disease may contribute to cardiovascular conditions (e.g., atherosclerosis and hypertension) through systemic inflammation and bacterial mechanisms, while also linking it with diabetes and metabolic syndrome due to shared inflammatory pathways.
- Periodontitis has also been implicated in conditions like chronic obstructive pulmonary disease, pneumonia, rheumatoid arthritis, chronic kidney disease and Alzheimer's, largely due to its role in systemic inflammation and potential microbial translocation.

**Supplementary Information:**

Zusatzmaterial online: Zu diesem Beitrag sind unter 10.1038/s41415-025-8642-2 für autorisierte Leser zusätzliche Dateien abrufbar.

## Introduction

Periodontal disease, one of the most prevalent oral health conditions worldwide, is an infecto-inflammatory disorder that affects the supporting structures of the teeth, including the gingivae and alveolar bone. It ranges from gingivitis, characterised by inflammation of the gingivae without destruction of the periodontal support tissues (cementum, periodontal ligament and bone), to periodontitis, a more severe condition characterised by the destruction of the supporting tissues.

The detrimental effects of this oral disease are not confined solely to the oral cavity. Numerous studies have demonstrated the correlation between periodontal disease and a variety of systemic conditions. This narrative review explores and highlights the evidence for the associations between periodontal disease and systemic diseases to provide a comprehensive understanding of the possible rationale behind their interaction, according to each bodily system. 

## Cardiovascular system

### Atherosclerosis and coronary artery disease

Translocated oral microbiota can directly or indirectly induce systemic inflammation that contributes to the pathogenesis of atherothrombogenesis and cardiovascular disease (CVD).^1^^,^^2^ Consequently, the connection between periodontal disease and CVD, including atherosclerosis, coronary artery disease and stroke, has been one of the most extensively studied relationships.^3^^,^^4^ The link between periodontitis and endothelial dysfunction (ED), a precursor to vascular diseases, can be potentially explained by three primary hypotheses: bacteriological, inflammatory and immunological.^5^

The bacteriological hypothesis suggests that periodontal pathogens, such as *Porphyromonas gingivalis*, enter the bloodstream, triggering an inflammatory response in endothelial cells.^6^^,^^7^ These bacteria have been detected in atherosclerotic plaques and their invasive ability is thought to promote ED by increasing pro-inflammatory mediators and adhesion molecules that contribute to atherosclerosis.^5^^,^^8^^,^^9^^,^^10^ The inflammatory theory proposes that mediators from diseased periodontium, such as IL-1β and TNF-α, enter the bloodstream and trigger systemic inflammation, including the production of acute-phase proteins.^11^ This systemic inflammatory response ultimately impairs endothelial function.

The immune hypothesis proposes that atypical immune responses can lead to increased tissue damage, particularly through a ‘hyperresponsive' phenotype that triggers heightened inflammation upon toll-like receptor (TLR) stimulation.^12^^,^^13^ This amplified response is thought to increase the risk of periodontitis and exacerbate ED due to excessive production of pro-inflammatory mediators. An alternative immunological theory proposes that an autoimmune response targets heat-shock proteins (HSPs).^7^ The immune system, primed by HSPs from periodontal pathogens like *P. gingivalis*, may crossreact with similar mammalian proteins in gingival connective tissue.^14^ This mechanism has also been associated with the development of atherosclerosis.

### Hypertension

Studies suggest a potential link between periodontal disease and hypertension,^15^^,^^16^^,^^17^ which may be partly due to elevated levels of inflammatory markers, such as C-reactive protein (CRP), fibrinogen and white blood cells associated with periodontal conditions.^18^^,^^19^ These inflammatory markers are known contributors to hypertension and coronary heart disease.^20^^,^^21^^,^^22^^,^^23^^,^^24^ Furthermore, it has been demonstrated that CRP can predict hypertension independently of baseline blood pressure and other traditional risk factors.^24^

Periodontitis can contribute to the overall inflammatory burden in individuals, leading to elevated CRP levels and, subsequently, an increased risk of CVD and hypertension.^25^ Gingival bleeding, indicative of periodontal inflammation, is consistently associated with increased systolic blood pressure and an elevated risk of developing hypertension.^15^ While severe periodontitis appears to be linked to a greater risk of hypertension, definitive conclusions regarding the causal relationship between periodontal disease and this condition remain elusive.^17^

### Clinical implications

Studies have shown that periodontal therapy, such as subgingival debridement, improves vascular function and reduces systemic inflammation, underscoring its potential value in mitigating cardiovascular risks.^6^ Randomised controlled trials (RCTs) have shown that periodontal treatment significantly reduces pathogenic microorganisms in dental plaque and systemic levels of IL-6, CRP and E-selectin, while also improving blood pressure, endothelial function and lipid profiles.^26^^,^^27^^,^^28^^,^^29^ Periodontal treatment initially causes acute systemic inflammation and endothelial dysfunction, but six months later, improvements in oral health have been associated with enhanced endothelial function.^26^ However, very low-quality evidence is available, rendering it insufficient to determine whether periodontal therapy can prevent the long-term recurrence of CVD in patients with periodontitis and there is no evidence for primary prevention.^30^

## Endocrine system

### Diabetes mellitus

The bidirectional relationship between diabetes mellitus (DM) and periodontal disease is well-established.^31^^,^^32^^,^^33^ Poorly controlled diabetes increases the risk of periodontal disease, while periodontitis contributes to poor blood glucose control.^34^ Type 2 diabetes frequently arises from systemic inflammation, which can compromise pancreatic β-cell functionality, induce cellular apoptosis and precipitate insulin resistance.^31^ Research indicates that this inflammatory response, as evidenced by biomarkers of acute-phase reaction and oxidative stress, may be intensified by the introduction of periodontal pathogens into the bloodstream, thereby elucidating a potential pathophysiological link between periodontitis and diabetes.^31^

Central to this association are the contributions of systemic dysregulation of advanced glycation endproducts and their receptor, along with various oxidative stress pathways on the periodontium.^31^ Moreover, both diabetes and periodontitis exhibit elevated levels of inflammatory markers, including IL-1β, TNF-α, IL-6 and the ratio of receptor activator of nuclear factor-kappa B ligand (RANKL) to osteoprotegerin,^35^ which may have an influence on the synergy of the disease, as well as a noted increase in oxidative stress and an upregulation of TLR-2 and TLR-4 in the context of both diseases, further reinforcing their interrelationship.^35^

### Metabolic syndrome

Metabolic syndrome is a group of conditions characterised by obesity, dyslipidaemia, hypertension and dysglycaemia, which collectively raise the risk of diabetes and CVD.^36^ Interest in the relationship between periodontitis and metabolic syndrome has been increasing, as both conditions are associated with prevalent non-communicable chronic inflammatory diseases.^37^ Like diabetes and CVD, metabolic syndrome has been associated with periodontitis.^19^^,^^37^^,^^38^^,^^39^ There is evidence indicating that metabolic syndrome and diabetes may influence the oral microbiome.^37^ However, it remains unclear whether the relationship between metabolic syndrome and periodontal disease is one-directional or bidirectional.^39^

Insulin resistance elevates pro-inflammatory cytokines, such as IL-6 and TNF-α, alongside reactive oxygen species (ROS), which can collectively drive systemic inflammation.^40^^,^^41^ This inflammatory state can activate matrix metalloproteinases (MMPs) and, in combination with ROS, may lead to periodontal destruction.^42^ Furthermore, inflammatory mediators prevalent in diabetic patients with periodontitis can lead to the upregulation of the osteoprotegerin/RANKL system, which plays a crucial role in regulating osteoclastogenesis and bone resorption.^43^ This dysregulation can disrupt bone homeostasis and potentially contribute to compromised periodontal health.^43^

Obesity may further exacerbate these effects by raising levels of pro-inflammatory cytokines and ROS, heightening the inflammatory response and accelerating periodontal breakdown.^44^ Additionally, high-fat diets have been shown to promote osteoclastogenesis, RANKL elevation and alveolar bone loss *in vivo*.^45^ Therefore, the combination of obesity, dyslipidemia and dysglycaemia in metabolic syndrome may create a synergistic effect, elevating inflammation and increasing the risk of periodontal disease. However, the exact mechanisms underlying the association between these two conditions remain unclear and continue to be the subject of ongoing research.

### Clinical implications

Evidence consistently shows that managing periodontal disease can enhance glycaemic control in diabetic patients, while poorly controlled diabetes tends to accelerate periodontal disease progression. Multiple studies, many of which are RCTs, have consistently reported reductions in HbA1c levels following periodontal therapy.^31^ Two Cochrane reviews, published in 2010 and 2015, confirmed statistically significant short-term reductions in HbA1c of approximately 3-4 mmol/mol (0.3-0.4%) within 3-4 months after treatment.^46^^,^^47^ More recently, a meta-analysis of RCTs also reported that non-surgical periodontal treatment led to statistically significant HbA1c reductions of about 4 mmol/mol (0.40%) at three months post-treatment.^48^ However, by six months, the reduction was lower.^48^ For individuals with metabolic syndrome, a holistic approach addressing both systemic and oral health issues is essential. Regular periodontal evaluations and treatments, combined with lifestyle modifications, can improve patient outcomes.

## Respiratory system

### Chronic obstructive pulmonary disease and pneumonia

Oral bacteria have been linked to hospital-acquired pneumonia through several mechanisms.^49^ First, dental plaque can harbour pulmonary pathogens, making the oral cavity a reservoir for respiratory infections like aspiration pneumonia in high-risk patients and patients with teeth or dentures.^50^^,^^51^^,^^52^^,^^53^ Second, enzymes associated with periodontal disease can alter mucosal surfaces, promoting respiratory pathogen adherence by degrading protective proteins like fibronectin. Third, these enzymes can destroy salivary defences, such as mucins, weakening non-specific host defences. Finally, in untreated periodontal disease, cytokines released from inflamed tissues may upregulate adhesion receptors, facilitating respiratory pathogen colonisation upon aspiration in vulnerable individuals.^49^^,^^54^^,^^55^

Reduced salivation and low salivary pH, common in ill or medicated patients, promote colonisation by respiratory pathogens.^53^ This colonisation is especially prevalent in institutionalised patients, such as those in hospital intensive care units (ICUs) or nursing homes, where poor oral hygiene is common.^56^

Recently, multiple systematic reviews have explored the link between periodontal health and respiratory diseases.^57^^,^^58^^,^^59^^,^^60^^,^^61^ These studies conclude that periodontal disease is associated with asthma, chronic obstructive pulmonary disease and pneumonia, which aligns with previous systematic reviews.^55^^,^^56^ However, these findings should be interpreted with caution, as other important covariates and confounding factors, such as smoking history and status, may be contributing to these results.

### Clinical implications

Oral interventions, including mechanical or chemical disinfection and antibiotic use, have been shown to reduce the incidence of nosocomial pneumonia by an average of 40%.^56^ Strong evidence (Grade A recommendation) indicates that improved oral hygiene and regular professional care significantly reduce the progression or occurrence of respiratory diseases in high-risk older individuals, particularly those in nursing homes and ICUs.^55^ Therefore, maintaining good oral hygiene, including regular professional cleaning, could help improve respiratory health, especially in vulnerable populations.

## Immune system

### Rheumatoid arthritis

The exact cause of rheumatoid arthritis (RA) remains unclear, but it is believed to arise from a combination of genetic and environmental factors that disrupt immune tolerance, particularly at mucosal surfaces.^62^^,^^63^ This disruption leads to the production of anti-citrullinated protein antibodies and rheumatoid factor (RF), contributing to joint inflammation and tissue damage through immune complex formation and pro-inflammatory cytokine release.^64^^,^^65^ Mikuls *et al.*^66^ demonstrated that *P. gingivalis* and periodontitis seem to influence the autoreactivity of RA, while also showing an independent association between periodontitis and established seropositive RA.

Periodontitis and RA exhibit several common features, including shared pathogenetic processes, cytokine profiles, inflammatory markers and genetic associations, such as HLA-DRB1.^67^ Both conditions are linked to polymorphisms in IL-1β and TNF-α, as well as the presence of citrullinated proteins and RF.^67^ Furthermore, these diseases share a pathobiology characterised by elevated pro-inflammatory cytokines, reduced tissue inhibitors of metalloproteinases, increased MMPs and prostaglandin E_2_ (PGE_2_), all of which contribute to the tissue destruction observed in periodontitis and RA.^68^

An antibody response to oral anaerobic bacteria have been identified within the synovial tissue and serum of RA patients, and oral bacterial DNA (deoxyribonucleic acid) has also been identified in their synovial fluid, suggesting a direct microbial connection.^67^ Notably *P. gingivalis* expresses peptidyl arginine deiminase, an enzyme that facilitates the citrullination of peptide antigens, a critical process in RA pathogenesis.^69^ Several systematic reviews have consistently demonstrated an association between periodontal health and RA.^70^^,^^71^^,^^72^^,^^73^^,^^74^

### Clinical implications

Clinical and biochemical markers suggest that non-surgical periodontal treatment may improve indicators of disease activity in RA. However, due to the small sample sizes and short follow-up periods (less than six months), larger longitudinal studies are necessary to better understand the relationship between these diseases and to evaluate the long-term effects of periodontal treatment on RA disease activity.^71^^,^^75^^,^^76^

## Renal system

### Chronic kidney disease

Chronic kidney disease (CKD) and periodontitis share several common risk factors, including DM, advancing age and smoking.^77^ Numerous studies have proposed a biological link between periodontitis and CKD, suggesting that periodontitis may serve as a non-traditional risk factor for CKD due to its potential to induce systemic inflammation through inflammatory mediators, such as IL-1β, IL-6, PGE_2_ and TNF-α.^77^^,^^78^ Additionally, bacteria and their byproducts can enter the bloodstream from periodontal tissues, further exacerbating the inflammatory response.^77^ Interestingly, periodontal treatments that reduce inflammation and bacterial load appear to enhance kidney function, providing additional support for this hypothesis.^79^

There is evidence supporting the positive association between periodontitis and CKD, along with the beneficial effects of periodontal treatment on estimated glomerular filtration rate.^77^ Fisher *et al.*^80^ demonstrated that periodontal disease is independently associated with CKD, even after accounting for both traditional and non-traditional risk factors. Furthermore, a significant link has been established between periodontitis and increased mortality rates in individuals with stages 3-5 CKD.^81^ A meta-analysis by Deschamps-Lenhardt *et al.*^78^ also confirmed a significant association between CKD and periodontitis, with the strength of this link increasing in cases of severe periodontitis (odds ratio = 2.39). The study concluded that periodontitis is associated with CKD even after multivariable adjustment. However, further research is needed to clarify the relationship between CKD and periodontitis.

### Clinical implications

Poor periodontal health and chronic periodontitis may contribute to systemic inflammation.^82^^,^^83^ This is especially important in CKD patients, as Gupta *et al*.^84^ demonstrated that increased inflammation is linked to decreased kidney function. Therefore, inflammation from periodontal disease could contribute to the progression of CKD by increasing levels of inflammatory markers like CRP. In patients with stage 3 and 4 CKD, CRP is an independent risk factor for all-cause mortality, including cardiovascular mortality.^85^ Therefore, managing periodontal disease and maintaining oral health through regular dental care could potentially reduce systemic inflammation and improve overall outcomes in CKD patients.

## Reproductive system

### Adverse pregnancy outcomes

Pregnancy can result in various adverse outcomes, such as low birth weight, preterm birth, growth restriction, pre-eclampsia, miscarriage and stillbirth. Maternal periodontitis may affect the foetal-maternal unit, both directly, by transmitting oral microorganisms to the unit, and/or indirectly, through inflammatory mediators that circulate and impact the unit. Elevated local and systemic inflammatory mediators, along with intrauterine infections, are commonly associated with these adverse pregnancy outcomes.^86^

Recent studies have drawn attention to the potential link between maternal periodontal disease and negative pregnancy outcomes, suggesting that inflammatory processes within the foetal-placental unit may play a role.^87^ While evidence indicates that maternal periodontitis is modestly associated with risks such as preterm delivery and low birth weight, the findings vary based on how periodontitis is defined.^87^ Furthermore, a preliminary study suggests that maternal periodontal disease and its progression may contribute to obstetric risks, such as preterm delivery, low birth weight and low weight for gestational age.^88^ However, despite extensive research, including nine systematic reviews, no consensus has been reached on the significance of any associations.^87^

### Clinical implications

A meta-analysis by Chen *et al.*^89^ revealed a high prevalence of periodontal disease during pregnancy, though the included studies showed significant heterogeneity. These findings align with research showing that the gingival index was significantly elevated during pregnancy compared to postpartum or non-pregnant women, without concommitant increase in plaque levels.^90^ Gil *et al.*^91^ also demonstrated that pregnancy increases CRP levels, which was positively associated with bleeding on probing and periodontal probing depths. However, after childbirth, both periodontal indices and CRP levels significantly decreased, along with a reduction in progesterone, without changes in plaque index (PI).^91^ The severity of periodontitis in pregnant women has been shown to be correlated with PI and toothbrushing frequency,^91^ highlighting the importance of good oral hygiene.

While periodontal therapy is safe and improves oral health in pregnant women with periodontitis, scaling and root surface debridement, with or without adjunctive antibiotics, has not been shown to improve pregnancy outcomes.^92^ When performing subgingival instrumentation during postpartum, it is important to carefully select local anaesthetic, as breastfeeding should be avoided for 48 hours after using certain agents, such as articaine.^93^ Given the potential link between periodontitis and adverse pregnancy outcomes, further research should explore different treatment strategies, including the timing and intensity of care.^86^

## Nervous system

### Alzheimer's disease

The accumulation of amyloid β plaques is a hallmark of Alzheimer's disease (AD); although, its causes remain poorly understood.^94^ It is suggested that periodontitis may contribute to the progression of AD through two primary mechanisms: elevated levels of pro-inflammatory cytokines and the invasion of cerebral tissues by microorganisms found in dental plaque biofilms.^95^ Kamer *et al.*^94^ found that after adjusting for relevant confounding factors, measures of periodontal disease were linked to amyloid accumulation in brain regions typically affected by amyloid deposits in AD patients, suggesting that periodontal inflammation or infection may elevate the risk of brain amyloid deposition.

Recent studies have highlighted a potential link between periodontal pathogens and AD in older adults, as well as an association between periodontitis and cognitive decline.^96^^,^^97^ Furthermore, a meta-analysis identified moderate to severe periodontitis as a significant risk factor for the development of dementia,^97^ suggesting that poor oral health may contribute to cognitive impairment in ageing populations. However, the literature and evidence exploring this relationship remains limited.

### Clinical implications

Parkinson's disease and dementia can have important implications on the management of periodontitis. Both conditions can affect motor skills, cognitive function and self-care abilities, which can lead to poor oral hygiene.^98^^,^^99^ Patients with Parkinson's often struggle with manual dexterity, making it difficult to perform effective toothbrushing or interdental cleaning, leading to plaque accumulation and increased risk of periodontitis. Similarly, those with dementia may forget or neglect oral hygiene routines, exacerbating periodontal issues. Additionally, the medications used to manage these neurodegenerative diseases may cause dry mouth, further increasing the risk of oral deterioration.^100^^,^^101^^,^^102^ Therefore, special consideration is required for the dental care of these patients, including tailored oral hygiene support, regular professional cleanings and caregiver involvement to help manage their oral health and prevent the progression of periodontitis and oral diseases.

## Conclusion

Periodontal disease is far more than a localised oral issue - it can have profound systemic implications, influencing conditions across cardiovascular, endocrine, respiratory and other bodily systems. The bidirectional relationship between oral health and overall wellbeing underscores the need for integrated care to reduce the burden of chronic diseases. By prioritising oral health, we can not only preserve dental function, but also positively impact systemic health. Future research and interdisciplinary collaboration are essential to fully discern these connections and optimise patient care across medical disciplines.

## Supplementary Information

Supplementary Information (PDF 122KB)

## Data Availability

The data included in the review is available from the authors on request and a full description of the methods is provided in the Supplementary Materials.
