# Supplementary material for: The interrelationship between periodontal disease and systemic health
Source: Br Dent J. 2025 Jul 25;239(2):103–8. doi: 10.1038/s41415-025-8642-2 (PMC12296551; doi:10.1038/s41415-025-8642-2)
Supplement: Supplementary file 1 — Supplementary Information (PDF 122KB) [file 41415_2025_8642_MOESM1_ESM.pdf]

Supplementary Materials

1. METHODS

This narrative literature review explores the association between periodontitis and systemic health by evaluating potential mechanisms explored in peer-reviewed research, focusing on the potential pathophysiological associations and clinical implications reported. The review only included articles published in English between 2000 and 2024, focusing on systematic reviews, meta-analyses, randomized control trials (RCTs), and observational studies.

A comprehensive literature search was conducted using electronic databases, including PubMed, ScienceDirect, and Google Scholar. The search included keywords such as *"Periodontitis," "Inflammation," "Microbiome," "Cardiovascular Diseases," "Atherosclerosis," "Coronary Artery Disease," "Hypertension," "Diabetes Mellitus," "Metabolic Syndrome," "Chronic Obstructive Pulmonary Disease," "Pneumonia," "Rheumatoid Arthritis," "Chronic Kidney Diseases," "Pregnancy Complications,"* and *"Alzheimer's Disease."*

This review focused on clinical, microbiological, and biological data, as well as experimental research exploring underlying mechanisms. Studies published in languages other than English, those without full-text availability, as well as abstracts, expert opinions, and letters were excluded.
